# Uptake of eye care services in South India: Retrospective mapping of self‐reported barriers using the Theoretical Domains Framework

**DOI:** 10.1111/opo.13424

**Published:** 2024-11-27

**Authors:** Vijay Kumar Yelagondula, Srinivas Marmamula, Rajashekar Varada, Ahalya Subramanian, John G. Lawrenson

**Affiliations:** ^1^ Brien Holden Institute of Optometry and Vision Sciences L V Prasad Eye Institute Hyderabad India; ^2^ Allen Foster Community Eye Health Research Centre, Gullapalli Pratibha Rao International Centre for Advancement of Rural Eyecare LV Prasad Eye Institute Hyderabad India; ^3^ School of Health & Medical Sciences City St George’s, University of London London UK; ^4^ Brien Holden Eye Research Centre LV Prasad Eye Institute Hyderabad India; ^5^ DBT Wellcome India Alliance LV Prasad Eye Institute Hyderabad India

**Keywords:** access to eye care, barriers to eye care, eye care, eye health seeking behaviour, theoretical domains framework

## Abstract

**Introduction:**

Understanding barriers to seeking eye care and providing evidence‐based theory‐informed solutions can improve the uptake of eye care services. Therefore, in this cross‐sectional study, we aim to report and analyse barriers to seeking eye care services among individuals with vision impairment in the Akividu region of Andhra Pradesh, India.

**Methods:**

Out of the 3000 enumerated participants, a total of 2587 were examined. All participants with vision impairment were asked to report barriers for not seeking eye care despite noticing reduced vision using a validated questionnaire. The reported barriers were mapped to the theoretical domains framework (TDF) to explore potential individual and environmental influences on the uptake of eye care services.

**Results:**

Barriers to seeking eye care services are most frequently mapped to the ‘beliefs about capabilities’, ‘environmental context and resources’ and ‘social influences’ domains of the TDF. The most frequently reported barrier was ‘aware of the problem but can manage’ (beliefs about capabilities), expressed by 43.4% (*n* = 156) and 55.7% (*n* = 337) of participants with distance and near vision impairment, respectively. ‘No one to accompany’ for an appointment (social influences) was a significant barrier for participants with distance vision impairment (*n* = 44, 12.2%) in comparison to participants with near vision impairment (*n* = 19, 3.1%). Additionally, fear of losing eyesight or operation or consultation (emotion) was a major deterrent for seeking eye care services, particularly among participants with distance vision impairment (*n* = 31, 8.6%) when compared with near vision impairment (*n* = 17, 2.8%).

**Conclusion:**

The uptake of eye care services is influenced by a complex set of interacting factors. Identification of potentially modifiable target behaviours provides an opportunity to develop theory‐informed solutions to improve uptake of services and prevent avoidable vision loss.

**Key points**

- This study enhances understanding of barriers to seeking eye care among adults with vision impairment by employing the theoretical domains framework.
- The findings emphasise the significant role of personal, environmental factors and social influences on eye care‐seeking behaviour.
- The study suggests that eye care providers should develop evidence‐based targeted interventions to improve the uptake of eye care services.

## INTRODUCTION

Globally, over a billion people have a vision impairment, with a significant proportion being treatable or avoidable.^1^ Despite global and national initiatives to reduce vision impairment, it continues to be a significant public health challenge, particularly in low‐ and middle‐income countries.^2^, ^3^ Health care‐seeking behaviour is crucial for the uptake of eye care services, but a range of interacting barriers, such as economic, socio‐cultural and personal barriers significantly impact service uptake.^2^, ^3^

The Grand Challenges in Global Eye Health prioritisation exercise identified 16 major global challenges, with three focusing on improving access to care and promoting equity.^4^ The report on these challenges highlighted the need to develop and implement eye care services that reach vulnerable groups, reduce out‐of‐pocket expenses and barriers to accessing services.^4^ In addition to availability, cost, awareness of services, attitudinal barriers (‘able to manage’) and social barriers (‘no one is willing to accompany’) also play a key role in determining the accessibility of eye care.^5^, ^6^, ^7^ Barriers can also vary with geographical location, population demographics and the eye condition under investigation.^7^, ^8^, ^9^

Despite initiatives by public and charitable organisations, untreated cataracts and uncorrected refractive errors continue to pose a significant challenge to reducing vision impairment in India.^10^, ^11^, ^12^ While previous studies have explored barriers to accessing eye care for various ocular diseases across different regions of India, they have not used a theory driven framework to get a better understanding of what influences behaviour.^7^, ^13^, ^14^, ^15^, ^16^ The theoretical domains framework (TDF) is gaining momentum and is widely used to report determinants of behavioural change holistically.^17^ The TDF was originally developed to understand the determinants of behaviour change among health care professionals in implementation research.^18^ It has also been used to understand patient behaviours in different health care settings.^19^ The determinants for seeking health care identified through the TDF can inform evidence‐based behaviour change techniques designed to enhance the uptake of health care services.^20^ However, only a limited number of studies have used a theory‐guided approach to study the determinants of uptake of eye care.^21^, ^22^, ^23^, ^24^, ^25^

LV Prasad Eye Institute, a World Health Organization (WHO) collaborating centre for the prevention of blindness, has been working towards reducing preventable vision impairment in South India and improving access to eye care for almost 35 years. The institute employs a pyramidal model of eye care delivery that offers primary, secondary and tertiary levels of eye care through a sustainable permanent infrastructure.^26^, ^27^ It is important to understand whether this model works in terms of overcoming barriers to accessing eye care services. A number of studies have been carried out, particularly in the region of Akividu in Andhra Pradesh to understand accessibility barriers, but these studies are over a decade old and need updating. In this study, we report on the barriers to seeking eye care services among individuals with distance and near vision impairments in Akividu, a region with access to primary, secondary and tertiary eye care centres. We used the TDF to understand better the factors influencing behaviour change, such as the uptake of eye care services and to move beyond traditional descriptive analyses of the barriers. The TDF also provides actionable insights into the specific domains which need to be addressed to facilitate behaviour change. Therefore, the aim of this study was to map eye care barriers to their respective TDF domains so as to identify determinants of behaviour and to signpost suitable behaviour change strategies to increase the uptake of eye care services.

## METHODS

The Akividu Vision Impairment Study (AVIS) protocol was approved by the Institutional Review Board of the Hyderabad Eye Research Foundation, Hyderabad, India. The study adhered to the tenants of the Declaration of Helsinki. Written informed consent was obtained from all participants before enrolling into the study.

The AVIS methodology has been published previously.^28^, ^29^ In brief, 3000 individuals aged 40 years and above were selected for the study using a multi‐stage cluster random sampling in the Akividu region. Three teams collected the data, each comprising a vision technician, a field investigator and a field worker. All study teams were trained on the study protocol. A study optometrist regularly visited each site to ensure quality control and to address any queries arising from the data collection. The clinical examination was conducted as per the Rapid Assessment of Visual Impairment (RAVI) protocol published elsewhere.^28^

In line with WHO definitions, distance vision impairment was defined as presenting visual acuity worse than 6/18 in the better eye.^1^, ^30^ Near vision impairment was defined as presenting binocular near visual acuity worse than N8 at a test distance of 40 cm.^30^ Both the distance and near vision impairment groups were mutually exclusive. The field investigator asked participants with distance and near vision impairment why eye care services were not sought despite having reduced vision using a validated questionnaire.^7^, ^31^ This questionnaire was initially developed and used in the Andhra Pradesh Eye Disease Study to report barriers to seeking eye care.^32^, ^33^ The reason for not seeking eye care services was asked in the local language (Telugu); participants’ responses were matched with a pre‐populated list of barrier responses, including other reasons in the data collection form.^7^, ^31^ In this study, the participant responses were matched to a predefined list of 12 barrier responses as listed in Table 1. If the barrier response did not match a category on the questionnaire, it was recorded under ‘other reasons’. In instances where multiple barriers were reported, participants were asked the primary reason for not seeking eye care, which was termed the ‘primary’ barrier. If the primary barrier was matched to other reasons, the participant was asked about the secondary barrier.

**TABLE 1:** Barriers reported by participants with distance vision impairment (DVI) and near vision impairment (NVI) to seeking eye care services.

Barriers	Participants with DVI (*n* = 359) *n* (%)	Participants with NVI (*n* = 604) *n* (%)
Aware of the problem, but can manage	156 (43.4%)	337 (55.7%)
Unaware of the problem	0 (0%)	52 (8.6%)
No one to accompany	44 (12.2%)	19 (3.1%)
Other health reasons	26 (7.2%)	16 (2.6%)
Services are not available or very far	0 (0%)	1 (0.1%)
Old age and need not felt	41 (11.4%)	35 (5.7%)
Fear of losing eyesight/Operation/Consultation	31 (8.6%)	17 (2.8%)
No time available/Other priorities	16 (4.4%)	44 (7.2%)
One eye adequate vision	11 (3.0%)	20 (3.3%)
Waiting for cataract to mature	5 (1.3%)	3 (0.5%)
Cannot afford consultation fee	3 (0.8%)	21 (3.4%)
Cannot afford cost of spectacles or surgery	15 (4.1%)	0 (0%)
Other reasons	11 (3.0%)	39 (6.4%)

Primary barriers (including other reasons) were quantified separately for participants with distance and near vision impairment. The barrier categories listed in the questionnaire were then mapped to the relevant domains of the TDF to quantify barriers based on potential determinants of behaviour. Initially, the first author (VKY) undertook the task of mapping the 12 pre‐identified barrier responses, including the actual responses recorded under ‘other’ reasons, to the corresponding domains of the TDF. This mapping process involved in‐depth discussions among team members (JGL, AS and SM) to ensure accuracy and relevance of the categorisation of barriers. Any discrepancies or disagreements in the mapping exercise were resolved through these discussions, leading to a consensus‐based final decision on the mapping of each barrier response to the relevant TDF domains.

### Statistical analysis

The study data were managed in a central database in Microsoft Access (Microsoft.com). Data analysis was performed using the Stata 14.0 software package (stata.com/stata14/). Descriptive statistics were used to report the results from the questionnaire and TDF mapping.

## RESULTS

A total of 2587 (86.2% response rate) participants were examined out of the 3000 enumerated for the study. The mean age ± SD of the total examined participants was 55.7 ± 11.4 years. Over half of the examined participants were women (*n* = 1406, 54.4%) and had no formal education (*n* = 1224, 47.3%). The prevalence of distance and near vision impairment was 12.8% (95% CI 11.5–14.1) and 27.1% (95% CI 25.2%–29.0%), respectively.^28^, ^29^ The mean age of the participants with distance vision impairment (66.0 ± 11.4 vs. 55.7 ± 11.3; *p* < 0.01) and near vision impairment (55.7 ± 10.9 vs. 54.0 ± 10.4; *p* < 0.01) was higher compared to those without visual impairment. Among the participants with distance (*n* = 359) and near vision impairment (*n* = 604), 57.6% and 57.4% were females, respectively. All participants (*n* = 963) with distance and near vision impairment responded to the survey questionnaire. Primary barriers to seeking eye care for participants with distance and near vision impairment (mutually exclusive groups) are shown in Table 1.

Among participants with distance vision impairment, 11 participants cited ‘other reasons’ (*n* = 11). Of these, only five participants had their actual responses recorded, while the remaining six participants’ responses were not recorded on the data collection form. Similarly, among the near vision impairment participants, 39 individuals cited ‘other reasons’. Of these, only 22 participants had their actual responses recorded in the data collection form. Therefore, the total barrier responses available for TDF mapping was 353 and 587 participants with distance and near vision impairment, respectively. The barriers mapped to the TDF domains are shown in Table 2. Barriers were most frequently mapped to the following TDF domains: ‘beliefs about capabilities', ‘environmental context and resources' and ‘social influences' (Table 3). A total of nine of 14 TDF domains were mapped. TDF domain definitions are provided in the Data S1.

**TABLE 2:** Barriers mapped to the theoretical domains framework (TDF).

Theoretical domains framework, domain names	Barriers mapped to domains
Beliefs about capabilities	Aware of the problem but can manage, old age and need not felt, other health reasons, one eye has adequate vision, not required near vision or glasses and eyes are fine
Environmental context and resources	Cannot afford consultation fee, cannot afford cost of spectacles or surgery, no time or other priorities, services are not available or very far and waiting for camp
Social influences	No one to accompany, doctor said vision would not recover, doctor said vision would not improve after surgery and family problems
Emotion	Fear of losing eyesight, operation or consultation
Knowledge	Unaware of the problem
Beliefs about consequences	Waiting for cataract to mature, no improvement even after surgery and using eye drops and comfortable
Intentions	Planning to go, taken appointment to visit hospital
Optimism	No use
Memory, attention and decision processes	Forgot to attend appointment

**TABLE 3:** Barriers to distance vision impairment (DVI) and near vision impairment (NVI) mapped to the theoretical domains framework.

Theoretical domains framework	Participants with DVI (*n* = 353) *n* (%)	Participants with NVI (*n* = 587) *n* (%)
Beliefs about capabilities	234 (66.2%)	419 (71.3%)
Social influences	46 (13.0%)	20 (3.4%)
Environmental context and resources	34 (9.6%)	67 (11.4%)
Emotion	31 (8.7%)	17 (2.8%)
Knowledge	NA	52 (8.8%)
Beliefs about consequences	6 (1.6%)	4 (0.6%)
Intentions	1 (0.2%)	7 (1.1%)
Optimism	1 (0.2%)	0 (0%)
Memory, attention and decision processes	0 (0%)	1 (0.17%)

Abbreviation: NA, not applicable.

The most salient TDF domain was ‘beliefs about capabilities’ (reported by 66.2% and 71.3% of participants with distance and near vision impairments, respectively). The common barriers within this domain were ‘aware of the problem but can manage’, ‘old age’ and ‘need not felt’. Economic barriers, including direct and indirect treatment costs and competing time demands, were the commonly reported barriers relating to the TDF domain ‘environmental context and resources’ (reported by 9.6% and 11.4% of those with distance and near vision impairments, respectively). ‘No one to accompany’ (social influences) was commonly reported by participants with distance vision impairment (12.2%) when compared to individuals with near impairment (3.1%). However, ‘unaware of the problem’ (knowledge) was reported by participants with near vision impairment alone. Less salient TDF domains included ‘belief about consequences’ and ‘emotion’. ‘Fear of losing eyesight or operation or consultation (emotion)’ and ‘waiting for cataract to mature’ (belief about consequences) were not major barriers to seeking eye care in this population. Overall, barriers to seeking eye care were mapped to nine of the 14 TDF domains.

## DISCUSSION

Universal Eye Health Coverage (UEHC) represents an equitable system where eye care services are accessible and affordable to all individuals without discrimination.^34^ Barriers to seeking eye care need to be investigated in different geographical locations to understand factors that might help or hinder the implementation of UEHC. To the best of our knowledge, this is the first study to categorise the barriers to seeking eye care among adults with vision impairment using the TDF. The key barriers to the uptake of eye care services in adults with vision impairment mapped to the TDF domains of ‘beliefs about capabilities’, ‘social influences’ and ‘environmental context and resources’.

‘Beliefs about capabilities’ was the most salient domain influencing eye care seeking behaviour among individuals with distance (66.2%) and near vision impairment (71.3%). Being ‘aware of the problem but can manage’ (43.4%) was the leading barrier that mapped to this domain in patients with distance vision impairment. These findings are similar to a previous study where being able to see adequately (69.4%) was the major reason why individuals who had no formal education and lived in a tribal region of Andhra Pradesh refused cataract surgery.^35^ Another important barrier that mapped to the ‘beliefs about capabilities’ domain was ‘old age and need not felt’ (reported by 11.4% of participants with distance vision impairment). However, this barrier was less commonly reported in Andhra Pradesh compared to the neighbouring state of Telangana, where over half the elderly participants over 60 years (63.5%) reported a lack of felt need, despite noticing a decrease in vision.^7^ This could be because of the difference in visual requirements, as participants in the Telangana study were older with a higher average mean age (67.7 ± 6.9 years). Therefore, they were less likely to be involved in active work. Among individuals with near‐vision impairment, being ‘aware of the problem but can manage’ (55.7%) was the leading barrier to seeking refraction services. These results were consistent with a study conducted in a rural Northern Indian population (58.7%).^15^

Among individuals with distance vision impairment, ‘social influences’ was the second leading TDF domain (13.0%) in determining eye care seeking behaviour. ‘No one to accompany’ (12.2%) was also a leading barrier that mapped to this domain. Similar findings were found in a study investigating a rural Chinese population, where the lack of family support was a major barrier (29.9%) to seeking low‐cost cataract surgery services.^5^ However, no one to accompany (2.9%) was not a major barrier reported in a previous study conducted between 1996 and 2000 in the state of Andhra Pradesh.^36^ No one to accompany was a major barrier in the present study, possibly because of a rise in nuclear families, with many working individuals having moved out of traditional joint‐family homes to find jobs elsewhere. Moreover, an increased life expectancy has led to many individuals who require eye care to also have mobility‐related issues, which necessitates additional support in getting to appointments when compared with previous studies.^37^, ^38^

The ‘environmental context and resources’ (9.6%) was the third most important domain influencing eye care seeking behaviour in those with distance vision impairment. ‘No time available’ or ‘other priorities’ (4.4%) and ‘cannot afford the cost of spectacles and surgery’ (4.1%) were the two (of five) major barriers that mapped to this domain. Financial barriers (4.9%) were not major determinants for seeking eye care in this study. In contrast, an investigation from Andhra Pradesh in 2007 found that ‘don't have money to pay for an eye check‐up’ (37%) was a major barrier among individuals with vision impairment.^36^ The study was conducted in the Krishna and West Godavari districts of Andhra Pradesh, which are financially stable districts with a much higher per capita income than the average for that state. A general rise in the economic strength of India in the past decade might also be a contributing factor.^39^ Cost is a major deterrent to seeking cataract surgery for many individuals in low‐ and middle‐income countries.^40^, ^41^ The ‘environmental context and resources’ were the second most salient domain associated with near vision impairment. The major individual barrier associated with this domain was ‘no time available or other priorities’ (7.2%).

None of the participants in this study reported the cost of spectacles as an issue, whereas the cost of spectacles was reported as a barrier to seeking near vision correction among the rural population in Northern India (16.7%).^15^ The cost of spectacles for near vision correction has also been described as a major barrier in studies carried out in Ghana (21%), rural Nigeria (39.3%) and Ethiopia (42.0%).^42^, ^43^, ^44^ In the current study population, the cost of consultation did not appear to be a barrier for individuals with distance vision impairment compared with near vision impairment. This might be due to an initiative by the Government of India called Vision 2020: the Right to Sight‐ India, which provides free cataract surgery in both non‐governmental organisations (NGOs) and government hospitals. Other government health initiatives, such as the Ayushman Bharat Pradhan Mantri Jan Arogya Yojna (PMJAY), allow individuals to access free eye care in existing primary health centres.^10^ In addition, many NGOs offer free or subsidised cataract surgery services, including the L V Prasad Eye Institute.^27^

‘Fear of surgery’ was identified as a barrier (8.7%) among participants with distance vision impairment, which mapped to the TDF domain of emotion. A report from South India found that ‘fear of surgery’ (1.8%) was an uncommon barrier among individuals over 40 years of age.^13^ Among the North Indian population, ‘fear of surgery’ (34%) and ‘fear loss of eye sight’ (33%) due to surgery were the major reasons for not seeking cataract surgery.^16^ In India, fear of surgery has not been reported consistently.^13^, ^16^ It is essential to investigate systematically the reasons behind this inconsistent reporting of fear related to seeking eye care services. Understanding these factors can help identify barriers to accessing care and ultimately increase the uptake of services by addressing the fear associated with seeking eye care. However, in a study from Ethiopia, ‘fear of cataract surgery complications’ (18.7%) was the leading barrier to seeking cataract surgery.^45^ To overcome ‘emotion’ related barriers the study authors recommended increasing the quantity and quality of cataract campaigns and using patients with good surgical outcomes as motivators for others to have surgery.^16^, ^45^ However, the literature provides limited evidence, and the only behaviour change technique that was mapped to the emotional domain of the TDF was the reduction of negative emotions.^46^ ‘Unaware of the problem’ (8.6%), which mapped to the TDF domain of ‘knowledge’, was a barrier to seeking near vision correction services. However, ‘unaware of the problem’ was the dominant barrier in a North Indian rural population (23.3%), Ghana (22%), Nigeria (23.4%) and Ethiopia (63.9%).^15^, ^42^, ^43^, ^44^ Barriers to seeking eye care services are often influenced by a range of interrelated factors, reflecting the complex nature of challenges that individuals face. For example, financial burden often results in postponing seeking eye care services as individuals prioritise other essential needs over eye health. For individuals with financial difficulties, the costs associated with surgery and transportation (environmental context and resources) can be a significant barrier. Moreover, if patients require someone to accompany them (social influences), this adds another layer of complexity to their situation. This need for social support not only impacts their willingness to seek care but also amplifies the financial burden.

A key strength of the TDF is that it provides a theoretical lens to identify influences on behaviour and facilitate the development of theory informed intervention strategies.^47^ Behaviour change techniques that address specific barriers in terms of TDF‐domains have been identified and are available via the online Human Behaviour Change Project‐Theory and Techniques Tool.^46^ This tool clarifies the behaviour change techniques that may be best suited to address particular TDF‐informed barriers (and which are not well suited or have inconclusive links). This approach provides a basis for selecting the behaviour change techniques that should be prioritised in intervention development. The most frequently reported barriers mapped to the TDF domain ‘beliefs about capabilities’. The behaviour change techniques that have the strongest link to this domain include ‘verbal persuasion about capability’ and ‘problem solving’. Interventions that prompt analysis of factors influencing their behaviour and the development of strategies to overcome these barriers are more likely to be successful.^46^

Major strengths of this study include the large population based representative sample size, and the fact that the study findings can potentially be generalised to other regions in India with a similar demographic profile. A methodologically robust approach was used, including a validated questionnaire, which is a quick and cost‐effective way to determine the frequency of barriers to seeking eye care, combined with the mapping of barrier responses to TDF domains to provide a theory‐informed and replicable strategy to understand behaviours. Future studies can map all identified TDF domains to suggest suitable behaviour change techniques using a theory‐based approach to increase the uptake of eye care services.^46^

## CONCLUSION

Eye health‐seeking behaviour in the Akividu region of India is influenced by a complex set of interacting factors. This study successfully mapped a single‐questionnaire barrier response to the TDF. Future work, using in‐depth qualitative interviews, will provide a deeper understanding of these barriers to confirm potential behavioural targets that could be incorporated into interventions to address modifiable barriers and enhance enablers to seeking eye care.

## AUTHOR CONTRIBUTIONS

**Vijay Kumar Yelagondula:** Conceptualization (equal); data curation (equal); formal analysis (lead); investigation (equal); methodology (equal); writing – original draft (lead); writing – review and editing (equal). **Srinivas Marmamula:** Conceptualization (lead); data curation (lead); formal analysis (equal); investigation (lead); methodology (lead); writing – original draft (equal); writing – review and editing (equal). **Rajashekar Varada:** Conceptualization (equal); data curation (equal); formal analysis (equal); investigation (equal); methodology (equal); writing – original draft (equal); writing – review and editing (equal). **Ahalya Subramanian:** Conceptualization (equal); formal analysis (equal); methodology (equal); writing – original draft (equal); writing – review and editing (equal). **John G. Lawrenson:** Conceptualization (equal); formal analysis (equal); methodology (equal); writing – original draft (equal); writing – review and editing (equal).

## FUNDING INFORMATION

The study was funded by Hyderabad Eye Research Foundation, L V Prasad Eye Institute (LEC08173).

## CONFLICT OF INTEREST STATEMENT

None of the authors has any potential conflict of interest in this study.

## Supporting information


Data S1:

## Data Availability

No additional data are available to share.
