# Supplementary material for: Uptake of eye care services in South India: Retrospective mapping of self‐reported barriers using the Theoretical Domains Framework
Source: Ophthalmic Physiol Opt. 2024 Nov 27;45(2):450–7. doi: 10.1111/opo.13424 (PMC11823384; doi:10.1111/opo.13424)
Supplement: Supplementary file 1 — Data S1: [file OPO-45-450-s001.docx]

| **Theoretical Domains Framework Domain names** | **Definitions** |
| --- | --- |
| 1.Belief about Capabilities  ( Beliefs, Self-confidence, perceived competence) | The acceptance of reality or ability of the individuals which influences eye care seeking behaviour. |
| 2. Environmental Context & Resources  (Personal and environmental stressors) | Environmental factors that influence an individual’s ability to seek eye care in a given environment. These resources include things like cost, physical infrastructure, access and organizational support. |
| 3. Social Influences  (Social norms, pressure and support) | Interpersonal relations that influence the individuals eye health seeking behaviour like belief in health care personnel and family and friends. |
| 4. Emotion  (Fear and anxiety) | Emotions such as fear, frustration, or enthusiasm influence one's motivation to seeking eye health. |
| 5. Knowledge  (Knowledge of the  condition, procedural knowledge) | Awareness and understating of the existing condition and treatment that influences eye health seeking behaviour. |
| 6. Beliefs about Consequences  (Outcome expectancies, beliefs) | Individual’s perceptions of the existing eye condition and treatment outcomes influencing eye health seeking behaviour. |
| 7. Intentions  (Stability of intentions, commitment) | Individuals commitment or conscious decision which influences eye health seeking behaviour. |
| 8. Optimism  (Optimism, pessimism) | Individual’s positive confidence in the expectations treatment outcomes influences eye health seeking behaviour. |
| 9. Memory, Attention and Decision Processes  (Cognitive overload and decision making) | Individuals cognitive abilities influence eye health seeking behaviour. |
| 10. Skills  (Ability or interpersonal skills) | Individual ability to access and utilize eye care services like ability to recognize symptoms and following the treatment instructions. |
| 11. Social professional role and identity  Social and professional identity | Individuals self-perception about the condition that influences eye health seeking behaviour. |
| 12. Reinforcement  Rewards and contingencies | Individuals eye health seeking behaviour is influenced by rewards, reminders and feedback. |
| 13. Goals  Goal setting and priority | Individuals keep specific target for seeking eye care, this goal intern drives the eye health seeking behaviour. |
| 14. Behavioural regulation  Self-monitoring and action planning | Individual uses different strategies and methods to control their actions to seek eye care services. |

Table: Theoretical Domains Framework: Definitions
